# Suspected acute exacerbation of idiopathic pulmonary fibrosis as an outcome measure in clinical trials

**DOI:** 10.1186/1465-9921-14-73

**Published:** 2013-07-13

**Authors:** Harold R Collard, Eric Yow, Luca Richeldi, Kevin J Anstrom, Craig Glazer

**Affiliations:** 1 University of California San Francisco, 505 Parnassus Avenue, San Francisco, CA, USA; 2 Duke Clinical Research Institute, 2400 Pratt Street, Durham, NC, USA; 3 University Hospital of Modena, Via del Pozzo 71, Modena, Italy; 4 University of Texas Southwestern, 5323 Harry Hines Blvd, Dallas, TX, USA

**Keywords:** Clinical trials, Endpoints, Acute exacerbation, Pulmonary fibrosis

## Abstract

**Background:**

Acute exacerbation of idiopathic pulmonary fibrosis has become an important outcome measure in clinical trials. This study aimed to explore the concept of suspected acute exacerbation as an outcome measure.

**Methods:**

Three investigators retrospectively reviewed subjects enrolled in the Sildenafil Trial of Exercise Performance in IPF who experienced a respiratory serious adverse event during the course of the study. Events were classified as definite acute exacerbation, suspected acute exacerbation, or other, according to established criteria.

**Results:**

Thirty-five events were identified. Four were classified as definite acute exacerbation, fourteen as suspected acute exacerbation, and seventeen as other. Definite and suspected acute exacerbations were clinically indistinguishable. Both were most common in the winter and spring months and were associated with a high risk of disease progression and short-term mortality.

**Conclusions:**

In this study one half of respiratory serious adverse events were attributed to definite or suspected acute exacerbations. Suspected acute exacerbations are clinically indistinguishable from definite acute exacerbations and represent clinically meaningful events. Clinical trialists should consider capturing both definite and suspected acute exacerbations as outcome measures.

## Background

Idiopathic pulmonary fibrosis (IPF) is a chronic, progressive, fibrotic lung disease with a median survival of approximately 3 years [1]. The natural history of disease is unpredictable, with many patients experiencing periods of relative stability punctuated by episodes of acute worsening [2,3]. Many of these acute worsenings are of unknown cause despite careful clinical evaluation. Idiopathic acute worsenings are called acute exacerbations of IPF and are associated with substantial morbidity and mortality [4]. Because of its clinical significance, acute exacerbation of IPF has become a key endpoint in clinical trials of novel drug agents for IPF.

Epidemiological data suggest an annual incidence per patient year of acute worsening in IPF of approximately 0.23, with acute exacerbation of IPF constituting slightly over half of acute worsening events [5]. However, incidence rates for acute exacerbation reported in recent clinical trial populations have been substantially lower [6-8]. The reason for this discrepancy is unknown.

Acute exacerbation of IPF is defined by strict clinical criteria, which require an acute onset of symptoms, new high-resolution computed tomography (HRCT) scan findings, and no evidence of infection or other identifiable cause [4]. Cases that are of unknown cause but do not fulfill the criteria listed due to missing data have been termed suspected acute exacerbations [4] Suspected acute exacerbations have generally been discounted in clinical studies, and the clinical features and behavior of this population are unknown.

In this study, we use a well-characterized cohort of patients with IPF enrolled in the Sildenafil Trial of Exercise Performance in IPF (STEP-IPF) to examine the incidence and clinical characteristics of suspected acute exacerbations of IPF. We find that suspected acute exacerbations are common in this clinical trial population and represent clinically important outcomes.

## Methods

Subjects included in this study were participants in the Sildenafil Trial of Exercise Performance in IPF (STEP-IPF) trial conducted by the IPF Clinical Research Network (IPFnet) [9]. Briefly, patients were eligible for enrollment in STEP-IPF if they met consensus criteria for the diagnosis of IPF [10] and had a diffusion capacity for carbon monoxide (DLCO) of less than 35% of the predicted value. Enrolled subjects were randomized to active drug (sildenafil 20 mg three times daily) or placebo for the first 12 weeks of the study, then given open label sildenafil for the second 12 weeks (Additional file 1: Figure E1). All STEP-IPF subjects provided informed consent for the use of their data in subsequent data analyses and were included in this analysis.

Subjects in STEP-IPF who experienced a respiratory serious adverse event during the course of the study (as determined by the site principal investigator) were considered acute respiratory worsenings for the purposes of the current study. As part of the STEP-IPF trial, possible acute exacerbation events were pre-specified as serious acute events and study sites were asked to collect detailed information about these events from the treating physician (not always at the study site). STEP-IPF did not identify definite and suspected acute exacerbation as specified by the IPFnet investigators in a perspective piece published after STEP-IPF was designed [4], so the original STEP-IPF adjudication results for acute exacerbation were not used in this analysis. All acute respiratory worsenings were reviewed independently by three of the authors (HC, CG, LR) and categorized as definite acute exacerbation suspected acute exacerbation, or other acute worsening [4]. Briefly, acute exacerbation was defined by acute onset of symptoms (<30 days in duration), new radiographic abnormalities (bilateral ground glass or consolidation on HRCT), and the absence of an identified infectious or alternative etiology (Additional file 2: Table E1). Suspected acute exacerbation of IPF was defined as an idiopathic acute respiratory worsening that could not be classified as a definite acute exacerbation due to missing data or criteria [4]. In cases considered as other acute worsenings, a cause was specified were possible (e.g. lower respiratory tract infection, pulmonary embolism). After individual reviews, all events were discussed as a group, and in cases of disagreement, a consensus result for each was determined. Inter-rater reliability for definite acute exacerbation and definite/suspected acute exacerbation was determined for the three reviewers by calculating single kappa statistics.

Comparisons between cases and selected controls were performed using the two-sample *t*-test or Wilcoxon rank sum and results were reported as mean (standard deviation), median (25^th^, 75^th^), or number (percent). Incidence rates per patient-year were determined for selected acute worsening subgroups (e.g. definite acute exacerbation, suspected acute exacerbation). Chi-square tests were used to determine the concordance of definite and suspected acute exacerbation with other measures of disease progression: change in forced vital capacity (FVC, defined as ≥10% relative decline), DLCO (defined as ≥15% relative decline), 6 minute walk distance (6MWD, defined as ≥30 meter decline), University of California San Diego Shortness of Breath Questionnaire (UCSD SOBQ, defined as ≥5 point increase) and St. George’s Respiratory Questionnaire (SGRQ, defined as ≥5 point increase). In events with missing data due to death, disease progression was considered to have occurred by the above variables. Survival analysis was performed using Kaplan-Meier survival estimates from the documented date of the acute worsening event. In some instances, definite acute exacerbation of IPF and suspected acute exacerbation of IPF events were combined into a single “acute exacerbation of IPF” group to enable statistical comparisons. All statistical analysis was performed using SAS Version 9.

## Results

### Cohort description

**Figure 1:**
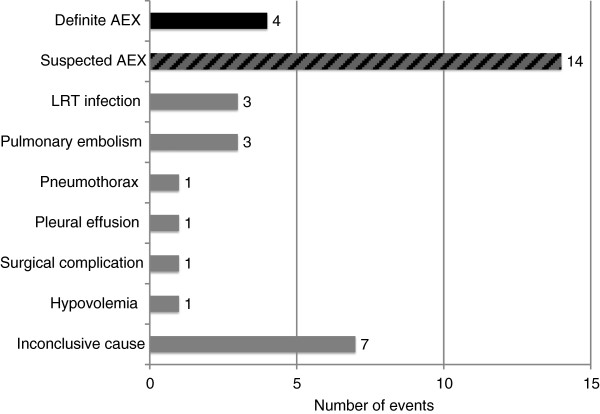
**Incidence of acute respiratory worsening by cause.** The adjudicated cause of the 35 acute respiratory worsening events in the STEP-IPF cohort are represented. Four definite acute exacerbations (black bar) and fourteen suspected acute exacerbations (striped bar) were identified. Cases of other acute worsenings are listed by cause (grey bars). Abbreviations: AEX = acute exacerbation; LTR = lower respiratory tract.

One hundred and eighty subjects constituted the study cohort. Thirty-five respiratory serious adverse events occurred during the course of the study; 31 of these were associated with hospitalization. Of these, eighteen (51%) were considered definite (four) or suspected (fourteen) acute exacerbation of IPF (Figure 1). Ten of the eleven original cases of acute exacerbation of IPF in the STEP-IPF study adjudicated per protocol and reported in the original manuscript were included in this group; the eleventh case was reclassified as other acute worsening. All but one of the fourteen suspected acute exacerbation cases were so classified because they did not undergo HRCT and/or respiratory culture; four were missing only evaluation for infection (Additional file 2: Table E2). High resolution computed tomography and respiratory culture were not required by the study protocol and were not routinely performed by treating physicians.

Inter-rater reliability for the adjudication of events as definite acute exacerbation of IPF (i.e. definite acute exacerbation versus suspected acute exacerbation, other or insufficient data) was good (0.72, 95% confidence interval 0.54 to 0.90). There were eight cases that at least one of three reviewers classified as definite acute exacerbation. Of these, three cases were unanimous (all three reviewers called them definite), five were classified as definite or suspected acute exacerbation, and one was classified as definite acute exacerbation, suspected acute exacerbation, and insufficient data available to adjudicate. The inter-rater reliability for definite or suspected acute exacerbation versus other or insufficient data was fair (0.43, 95% confidence interval 0.26 to 0.60).

### Incidence and characteristics of acute exacerbation

**Figure 2:**
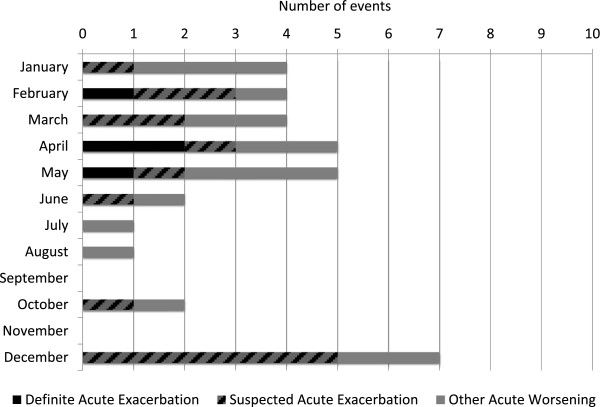
**Seasonal variation in risk of definite and suspected acute exacerbation and acute worsening.** The number of acute worsening events (definite acute exacerbation = black; suspected acute exacerbation = striped; other acute worsening = grey) are demonstrated by calendar month. The number of acute exacerbation events (definite and suspected) per month normalized to the number of patients at risk were significantly higher from December to May than from June to November, with an odds ratio of 8.06 (95% CI 1.76, 36.89, p = 0.007).

The overall incidence per patient year of acute worsening of any cause was 0.39 (CI 0.31, 0.60). The incidence of definite acute exacerbation of IPF was 0.04 (CI 0.01, 0.12) and of suspected acute exacerbation was 0.16 (CI 0.09, 0.26). There was a strong seasonal association, with an odds ratio of 8.06 (CI 1.76, 36.89, p = 0.007) for definite or suspected acute exacerbation of IPF occurring from December through May compared to June through November (Figure 2). This association was also true for other acute worsenings. This finding was not explained by differences in the number of patients at risk during those months.

Subjects with definite or suspected acute exacerbation of IPF reported increased dyspnea (94%) and cough (88%). Symptoms of infection such as fever and congestion were reported in the minority (31% and 24%, respectively). Over half of these subjects developed respiratory failure requiring non-invasive ventilation (18%) or intubation (35%). Most subjects received corticosteroids (81%) and antibiotic therapy (88%) as part of their treatment. Characteristics of definite and suspected acute exacerbations were identical, but the number of cases was too small in these groups to allow statistical comparisons.

### Predictors of acute exacerbation and acute worsening

Subjects with definite or suspected acute exacerbation of IPF had worse dyspnea score (UCSD SOBQ), worse pulmonary physiology (FVC and DLCO), worse 6MWD, and worse oxygenation at baseline compared to subjects without acute worsening (Table 1). There was also an increased prevalence of patient reported coronary artery disease compared to subjects without acute worsening (53% vs. 25%, p = 0.02). Subjects who developed definite or suspected acute exacerbation of IPF had significantly greater baseline prednisone use (41% vs. 19%, p = 0.05) and a trend towards lesser baseline PPI use (18% vs. 45%, p = 0.09). There was no significant difference between groups in study arm (active sildenafil vs. placebo), age, or history of cigarette smoking. Subjects with other acute worsenings appeared similar to the acute exacerbation subjects, and risk factors for acute worsening of any cause were similar to those identified for acute exacerbation.

**Table 1:** Demographics and baseline characteristics by acute worsening status

**Variable**	**Acute exacerbation**	**Other acute**	**No acute**	**Acute exacerbation vs. No**	**Any acute worsening vs. No**
	*N = 17	**worsening N = 13**	**worsening N = 150**	**acute worsening P-value**	**acute worsening P-value**
Age, years	71	69	69	0.27	0.30
Male gender	17 (100%)	11 (85%)	122 (81%)	0.08	0.11
Disease duration, years	2.01	2.97	1.86	0.74	0.19
Body mass index	28.3	29.3	29.0	0.64	0.75
Ever smoker	10 (59%)	11 (85%)	116 (77%)	0.13	0.39
HRCT UIP pattern	14 (82%)	13 (100%)	121 (81%)	>0.99	0.22
Biopsy proven disease	7 (41%)	5 (39%)	75 (50%)	0.80	0.76
6MWT distance, meters	179.8	208.5	279.6	<0.001	<0.001
FVC % predicted	49.7	54.7	57.8	0.03	0.04
FEV1/FVC ratio	0.77	0.77	0.77	0.45	0.63
DLCO % predicted	21.7	23.3	27.1	<0.001	<0.001
PaO2, mmHg	60.3	61.5	69.5	0.01	<0.001
UCSD score	57	60	45	0.03	0.003
SF-36 physical score	33.47	28.21	34.58	0.44	0.03
SF-36 mental score	50.87	49.20	51.71	0.83	0.43
SGRQ total score	55.64	64.60	51.80	0.28	0.01
Sildenafil use **	8 (47%)	5 (39%)	76 (51%)	0.78	0.46
PPI use	3 (18%)	7 (54%)	68 (45%)	0.09	0.45
Prednisone use	7 (41%)	2 (15%)	28 (19%)	0.05	0.01

* Combined definite and suspected cases.

** Subjects randomized to active therapy during the first 12 weeks of STEP-IPF.

### Prognostic significance of acute exacerbation and acute worsening

**Figure 3:**
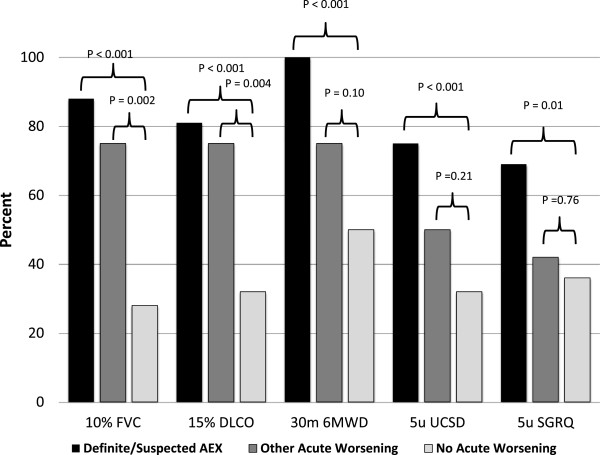
**Association of acute exacerbation and other acute worsenings with disease progression.** Disease progression was variably defined by ≥10% relative decline in forced vital capacity (FVC), ≥ 15% relative decline in diffusion capacity for carbon monoxide (DLCO), ≥ 30 meter decline in 6 minute walk distance (6MWD), ≥ 5 point increase in the University of California San Diego (UCSD) shortness of breath questionnaire, and ≥ 5 point increase in the St. George’s Respiratory Questionnaire (SGRQ).

**Figure 4:**
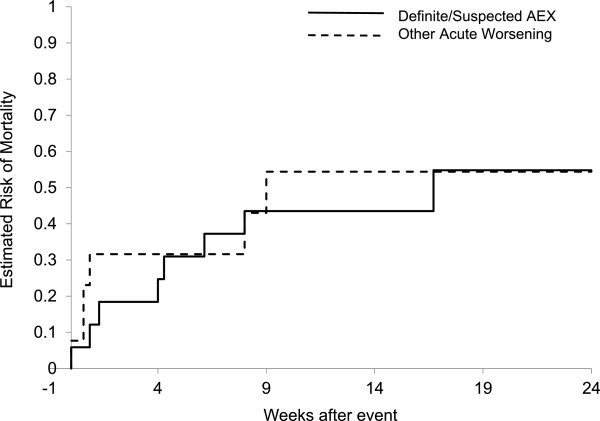
**Acute worsening and survival.** Cases adjudicated as definite or suspected acute exacerbation (AEX) of IPF (solid line) and other acute worsening (dashed line) were associated with similar short-term risk of death.

Definite or suspected acute exacerbation of IPF was associated with progression of disease as defined by categorical change in FVC, DLCO, 6MWD, UCSD SOBQ, and SGRQ (Figure 3). Findings were similar for subjects with other acute worsenings. There were fifteen deaths overall in the study cohort; eight (53%) occurred in the definite (n = 2) or suspected (n = 6) acute exacerbation of IPF group; six occurred in the other acute worsening group. Only one occurred in subjects without acute worsening. Overall 24-week mortality in the definite or suspected acute exacerbation of IPF group was 47%, similar to that of subjects with other acute worsenings (Table 2). This was significantly greater that the 24-week mortality in subjects without acute worsening (1%, p < 0.01). Most deaths in the definite or suspected acute exacerbation of IPF and other acute worsening group occurred within twelve weeks of the acute exacerbation event (Figure 4).

**Table 2:** 24-week mortality by acute worsening status

**Group**	**Number of deaths**	**Number at risk**	**24-week mortality**
Acute Exacerbation of IPF *	8	17	47%
Other Acute Worsening	6	13	46%
No Acute Worsening	1	150	1%

* Combined definite and suspected cases. Two deaths were in the definite acute exacerbation group (50% mortality rate at 24 weeks) and six deaths were in the suspected acute exacerbation group (46% mortality rate at 24 weeks).

Abbreviations: *IPF*, idiopathic pulmonary fibrosis.

## Discussion

These results suggest that suspected acute exacerbations of IPF are common, clinically relevant events in patients enrolled in clinical trials of IPF. The similar clinical characteristics, risk factors, and clinical outcomes of definite and suspected acute exacerbation cases suggest that suspected acute exacerbations may, in many cases, represent true acute exacerbation of IPF. Based on these observations, we believe that suspected acute exacerbations should be captured and analyzed as a clinical trial endpoint.

Very few definite acute exacerbations of IPF were identified in this study, consistent with recent clinical trial results [6-8]. We hypothesize that the adoption of strict criteria for acute exacerbation of IPF and logistical limitations in the clinical trial setting (e.g. difficulty obtaining clinical reports from outside hospitals, adjudicating cases based only on written reports) has resulted in the under-recognition of actual acute exacerbation events. The reclassification of 6 acute exacerbations from the original STEP-IPF trial as suspected acute exacerbations in this reanalysis using the current, stricter criteria for acute exacerbation illustrates this point. We believe these “missed” acute exacerbations are captured by the suspected acute exacerbation endpoint. Interestingly, combining definite and suspected acute exacerbations in this STEP-IPF re-analysis results in an incidence of 0.20 per patient year, similar to what has been described in a recent large epidemiological study [5]. This is unlikely to be the only reason for the low number of definite acute exacerbations identified in this and other clinical trials (e.g. improved clinical care for subjects enrolled in trials could contribute), but the possibility of a methodological contribution should be explored further in future clinical trials.

Disease severity and ethnicity have been suggested as potential reasons for varying rates of acute exacerbation in clinical trials [5]. In the current study, the finding that multiple measures of disease severity are associated with increased risk of definite and suspected acute exacerbation suggests that patients with earlier disease (i.e. those patients typically enrolled into clinical trials of novel drug agents) may have a lower incidence than the general population of IPF patients and the more advanced population enrolled into STEP-IPF. However, there are plausible explanations for why acute exacerbation appears to occur more frequently in patients with advanced disease that have nothing to do with the actual rate of events (e.g. decreased physiologic reserve in advanced disease resulting in more clinical signs and symptoms, increased health care utilization in advanced disease resulting in a greater chance of clinical recognition). Ethnic and racial differences in acute exacerbation risk may also exist, although data from recent clinical trials conducted in the United States, Europe and Japan using similar criteria for acute exacerbation adjudication do not support this hypothesis [6,7,11-13].

We believe a strong argument can be made for including both definite and suspected acute exacerbations as outcome measures in clinical trials. First, suspected acute exacerbations of IPF are clearly common, clinically important events. Second, our data support the hypothesis that many suspected acute exacerbations are indeed acute exacerbations. Third, in many clinical centers, HRCT scanning and respiratory cultures are not routinely performed on IPF patients with idiopathic respiratory worsening, as the tests are not felt to change clinical management (patients are simply treated empirically for both pneumonia and acute exacerbation), and involve cost and risk. These cases, therefore, cannot be classified as definite acute exacerbations. For all of these reasons, we believe that including suspected acute exacerbations along with definite acute exacerbations as endpoints in clinical trials will most appropriately capture the clinically meaningful events of interest to clinical trialists. Whether definite and suspected acute exacerbations should in fact be combined into a single endpoint in clinical trials remains unclear.

The association of season with both definite and suspected acute exacerbation of IPF is notable, and suggests that there may be factors such as respiratory viral infection or air pollution (products of combustion, particulate matter) that contribute to the pathogenesis of these events. This seasonal association has not been reliably demonstrated in retrospective cohort studies [5,14], and it has been difficult to demonstrate widespread evidence of infection in cohorts of carefully evaluated cases [5,15,16]. However, this finding is striking and suggests that seasonal risk factors may play a role in acute exacerbation of IPF.

## Conclusion

In summary, we report that suspected acute exacerbations of IPF are common in the clinical trial setting, are clinically indistinguishable from definite acute exacerbations, and are associated with a similarly high risk of disease progression and short-term mortality. There should be no doubt that suspected acute exacerbation of IPF is a clinically important event in the lives of patients. We propose that those responsible for designing future clinical trials rethink the methodological approach to capturing acute exacerbation of IPF, and consider less restrictive criteria. Specifically, we propose recording both definite and suspected acute exacerbations of IPF, and analyzing them either as separate outcome measures or as a combined idiopathic acute worsening endpoint. Additional changes to the methodology of acute exacerbation identification in clinical trials may also be worthwhile (e.g. site-investigator-driven identification process vs. central adjudication), and studies comparing the appropriateness and feasibility of various approaches to defining and identifying acute exacerbation of IPF in clinical trials are greatly needed.

## Abbreviations

HRCT: High resolution computed tomography; UIP: Usual interstitial pneumonia; 6MWT: 6 minute walk test; FVC: Forced vital capacity; FEV1: Forced expiratory volume in 1 second; DLCO: Diffusion capacity for carbon monoxide; PaO2: Arterial partial pressure of oxygen; UCSD: University of California San Diego; SF-36: Short form 36; SGRQ: St. George’s respiratory questionnaire; PPI: Proton pump inhibitor.

## Competing interests

HC has competing interests with Biogen, Boehringer Ingelheim, FibroGen, Genentech, Genoa, Gilead, InterMune, MedImmune, and Promedior; EY reports no competing interests; LR has competing interests with Boehringer Ingelheim, InterMune, MedImmune, and Takeda; KA has competing interests with Abbott Vascular, AstraZeneca, Pfizer, University of North Carolina, and Vertex; CG reports no competing interests.

## Authors’ contributions

HC and CG conceived of the study; EY and KA performed all statistical analyses; all authors participated in the study design, data collection and interpretation, and drafting and revision of the manuscript. All authors read and approved the final manuscript.

## Supplementary Material

Additional file 1: Figure E1 Study design for STEP-IPF. Patients were eligible for enrollment in STEP-IPF if they met consensus criteria for the diagnosis of IPF and had a diffusion capacity for carbon monoxide (DLCO) of less than 35% of the predicted value. Enrolled subjects were randomized to active drug (sildenafil 20 mg three times daily) or placebo for the first 12 weeks of the study, then given open label sildenafil for the second 12 weeks. Click here for file

Additional file 2: Table E1 Diagnostic Criteria for Acute Exacerbation. **Table E2.** Reasons for “suspected acute exacerbation” diagnosis. Click here for file

## References

[B1] Raghu G Collard HR Egan JJ Martinez FJ Behr J Brown KK Colby TV Cordier JF Flaherty KR Lasky JA An official ATS/ERS/JRS/ALAT statement: idiopathic pulmonary fibrosis: evidence-based guidelines for diagnosis and management Am J Respir Crit Care Med 2011 183 788 824 10.1164/rccm.2009-040GL 21471066 PMC5450933

[B2] Ley B Collard HR King TE Jr Clinical course and prediction of survival in idiopathic pulmonary fibrosis Am J Respir Crit Care Med 2011 183 431 440 10.1164/rccm.201006-0894CI 20935110

[B3] Kim DS Collard HR King TE Jr Classification and natural history of the idiopathic interstitial pneumonias Proc Am Thorac Soc 2006 3 285 292 10.1513/pats.200601-005TK 16738191 PMC2658683

[B4] Collard HR Moore BB Flaherty KR Brown KK Kaner RJ King TE Jr Lasky JA Loyd JE Noth I Olman MA Acute exacerbations of idiopathic pulmonary fibrosis Am J Respir Crit Care Med 2007 176 636 643 10.1164/rccm.200703-463PP 17585107 PMC2094133

[B5] Song JW Hong SB Lim CM Koh Y Kim DS Acute exacerbation of idiopathic pulmonary fibrosis: incidence, risk factors and outcome Eur Respir J 2011 37 356 363 10.1183/09031936.00159709 20595144

[B6] Noble PW Albera C Bradford WZ Costabel U Glassberg MK Kardatzke D King TE Jr Lancaster L Sahn SA Szwarcberg J Pirfenidone in patients with idiopathic pulmonary fibrosis (CAPACITY): two randomised trials Lancet 2011 377 1760 1769 10.1016/S0140-6736(11)60405-4 21571362

[B7] King TE Jr Brown KK Raghu G du Bois RM Lynch DA Martinez F Valeyre D Leconte I Morganti A Roux S Behr J BUILD-3: a randomized, controlled trial of bosentan in idiopathic pulmonary fibrosis Am J Respir Crit Care Med 2011 184 92 99 10.1164/rccm.201011-1874OC 21474646

[B8] Taniguchi H Ebina M Kondoh Y Ogura T Azuma A Suga M Taguchi Y Takahashi H Nakata K Sato A Pirfenidone in idiopathic pulmonary fibrosis Eur Respir J 2010 35 821 829 10.1183/09031936.00005209 19996196

[B9] Zisman DA Schwarz M Anstrom KJ Collard HR Flaherty KR Hunninghake GW A controlled trial of sildenafil in advanced idiopathic pulmonary fibrosis N Engl J Med 2010 363 620 628 20484178 10.1056/NEJMoa1002110 PMC3587293

[B10] American Thoracic Society Idiopathic pulmonary fibrosis: diagnosis and treatment. international consensus statement. American thoracic Society (ATS), and the European respiratory society (ERS) Am J Respir Crit Care Med 2000 161 646 664 10673212 10.1164/ajrccm.161.2.ats3-00

[B11] King TE Jr Behr J Brown KK du Bois RM Lancaster L de Andrade JA Stahler G Leconte I Roux S Raghu G BUILD-1: a randomized placebo-controlled trial of bosentan in idiopathic pulmonary fibrosis Am J Respir Crit Care Med 2008 177 75 81 10.1164/rccm.200705-732OC 17901413

[B12] King TE Jr Albera C Bradford WZ Costabel U Hormel P Lancaster L Noble PW Sahn SA Szwarcberg J Thomeer M Effect of interferon gamma-1b on survival in patients with idiopathic pulmonary fibrosis (INSPIRE): a multicentre, randomised, placebo-controlled trial Lancet 2009 374 222 228 10.1016/S0140-6736(09)60551-1 19570573

[B13] Richeldi L Costabel U Selman M Kim DS Hansell DM Nicholson AG Brown KK Flaherty KR Noble PW Raghu G Efficacy of a tyrosine kinase inhibitor in idiopathic pulmonary fibrosis N Engl J Med 2011 365 1079 1087 10.1056/NEJMoa1103690 21992121

[B14] Olson AL Swigris JJ Raghu G Brown KK Seasonal variation: mortality from pulmonary fibrosis is greatest in the winter Chest 2009 136 16 22 10.1378/chest.08-0703 18689582 PMC3662208

[B15] Wootton SC Kim DS Kondoh Y Chen E Lee JS Song JW Huh JW Taniguchi H Chiu C Boushey H Viral infection in acute exacerbation of idiopathic pulmonary fibrosis Am J Respir Crit Care Med 2011 183 1698 1702 10.1164/rccm.201010-1752OC 21471095 PMC3136996

[B16] Huie TJ Olson AL Cosgrove GP Janssen WJ Lara AR Lynch DA Groshong SD Moss M Schwarz MI Brown KK Frankel SK A detailed evaluation of acute respiratory decline in patients with fibrotic lung disease: aetiology and outcomes Respirology 2010 15 909 917 10.1111/j.1440-1843.2010.01774.x 20546190

