# Supplementary figures and images for: Suspected acute exacerbation of idiopathic pulmonary fibrosis as an outcome measure in clinical trials
Source: Respir Res. 2013 Jul 13;14(1):73. doi: 10.1186/1465-9921-14-73 (PMC3729659; doi:10.1186/1465-9921-14-73)

## Slide 1
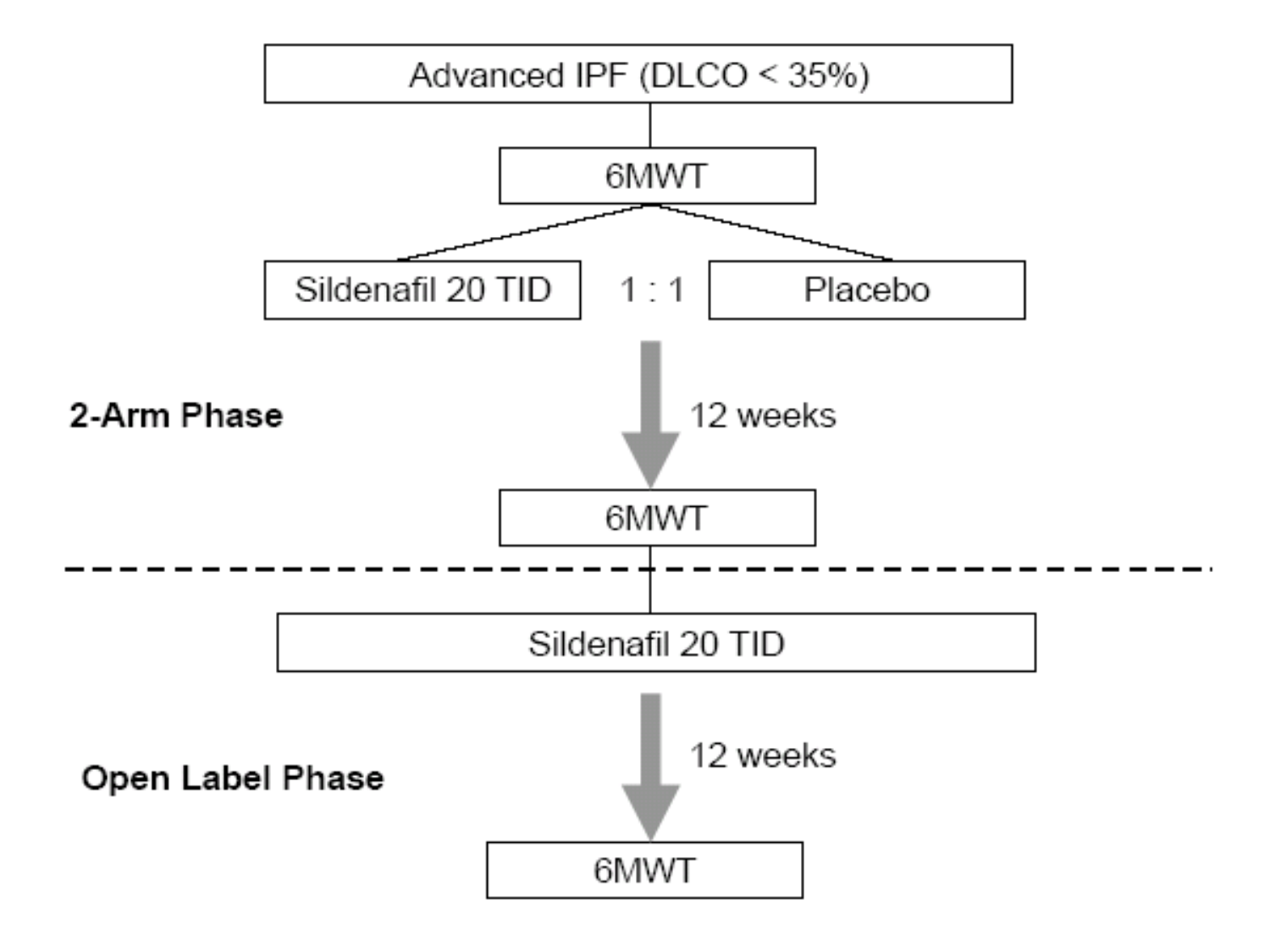

Supplement: Additional file 1: Figure E1 — Study design for STEP-IPF. Patients were eligible for enrollment in STEP-IPF if they met consensus criteria for the diagnosis of IPF and had a diffusion capacity for carbon monoxide (DLCO) of less than 35% of the predicted value. Enrolled subjects were randomized to active drug (sildenafil 20 mg three times daily) or placebo for the first 12 weeks of the study, then given open label sildenafil for the second 12 weeks. [file 1465-9921-14-73-S1.pptx]
