# Supplementary material for: Suspected acute exacerbation of idiopathic pulmonary fibrosis as an outcome measure in clinical trials
Source: Respir Res. 2013 Jul 13;14(1):73. doi: 10.1186/1465-9921-14-73 (PMC3729659; doi:10.1186/1465-9921-14-73)

**Online Data Supplement:**

**Suspected Acute Exacerbation of Idiopathic Pulmonary Fibrosis As An Outcome Measure in Clinical Trials**

Harold R Collard, MD

Eric Yow, MS

Luca Richeldi, MD, PhD

Kevin J. Anstrom, PhD

Craig Glazer, MD, MSPH

**Table E1: Diagnostic Criteria for Acute Exacerbation**

| Unexplained worsening or development of dyspnea within 30 days |
| --- |
| High-resolution computed tomography with new bilateral ground glass abnormality and/or consolidation superimposed on a background reticular or honeycomb pattern consistent with usual interstitial pneumonia pattern |
| No evidence of pulmonary infection by endotracheal aspirate of bronchoalveolar lavage |
| Exclusion of alternative causes including left heart failure, pulmonary embolism, or other identifiable cause of acute lung injury |

**Table E2: Reasons for “suspected acute exacerbation” diagnosis**


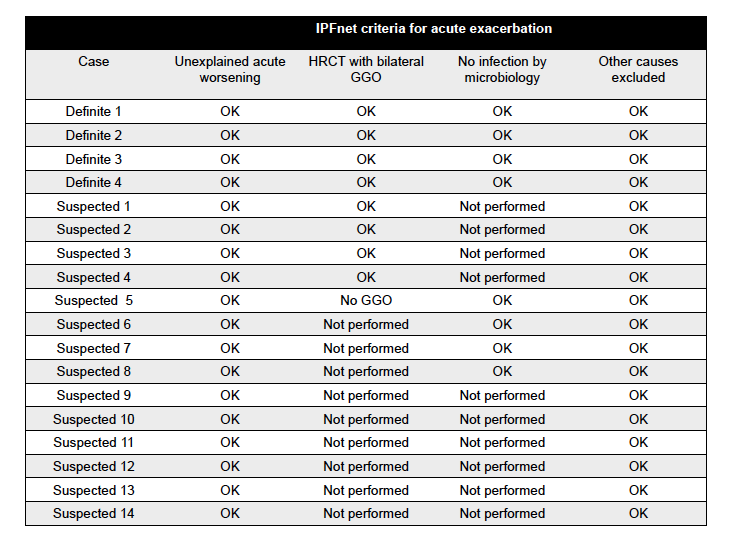

Supplement: Additional file 2: Table E1 — Diagnostic Criteria for Acute Exacerbation. Table E2. Reasons for “suspected acute exacerbation” diagnosis. [file 1465-9921-14-73-S2.docx]
